# Aerosols, airflow, and more: examining the interaction of speech and the physical environment

**DOI:** 10.3389/fpsyg.2023.1184054

**Published:** 2023-05-15

**Authors:** Caleb Everett, Chantal Darquenne, Renee Niles, Marva Seifert, Paul R. Tumminello, Jonathan H. Slade

**Affiliations:** ^1^Departments of Anthropology and Psychology, University of Miami, Coral Gables, FL, United States; ^2^Department of Medicine, University of California, San Diego, La Jolla, CA, United States; ^3^Department of Chemistry and Biochemistry, University of California, San Diego, La Jolla, CA, United States

**Keywords:** phonetics, environment, aerosols, airflow, adaptation, acoustic, respiratory

## Abstract

We describe ongoing efforts to better understand the interaction of spoken languages and their physical environments. We begin by briefly surveying research suggesting that languages evolve in ways that are influenced by the physical characteristics of their environments, however the primary focus is on the converse issue: how speech affects the physical environment. We discuss the speech-based production of airflow and aerosol particles that are buoyant in ambient air, based on some of the results in the literature. Most critically, we demonstrate a novel method used to capture aerosol, airflow, and acoustic data simultaneously. This method captures airflow data via a pneumotachograph and aerosol data via an electrical particle impactor. The data are collected underneath a laminar flow hood while participants breathe pure air, thereby eliminating background aerosol particles and isolating those produced during speech. Given the capabilities of the electrical particle impactor, which has not previously been used to analyze speech-based aerosols, the method allows for the detection of aerosol particles at temporal and physical resolutions exceeding those evident in the literature, even enabling the isolation of the role of individual sound types in the production of aerosols. The aerosols detected via this method range in size from 70 nanometers to 10 micrometers in diameter. Such aerosol particles are capable of hosting airborne pathogens. We discuss how this approach could ultimately yield data that are relevant to airborne disease transmission and offer preliminary results that illustrate such relevance. The method described can help uncover the actual articulatory gestures that generate aerosol emissions, as exemplified here through a discussion focused on plosive aspiration and vocal cord vibration. The results we describe illustrate in new ways the unseen and unheard ways in which spoken languages interact with their physical environments.

## 1. Background: effects of the environment on speech, and of speech on the environment

While our understanding of language and linguistic diversity continues to evolve, one area of research that remains underexplored is the interaction of speech and the physical environment. Like other facets of human behavior, languages are affected over the long-term by external physical factors (Bentz et al., 2018). Conversely, however, languages themselves might affect the immediate physical environments of their speakers, and this impact could in turn affect other individuals in those environments. In this paper, we dedicate some of our attention to exploring the way in which two articulatory gestures in languages appear to impact the physical environments of their speakers via differences in airflow and generation of aerosol particles. One of these gestures, vocal cord vibration, is critical to all spoken languages. The second, aspiration, is found in about a fifth of the world’s languages, including English. The exploration of the aerosol generation characteristics of these articulatory gestures is preliminary, serving primarily to illustrate a novel method we have developed for simultaneously capturing airflow, acoustic, and aerosol data. First, we briefly survey some of the research suggesting that languages are themselves affected by the physical environments in which they are spoken.

It is becoming increasingly clear that languages evolve in ways that are sensitive to the typical characteristics of their speakers’ environments. To cite one relatively obvious example, the frequency with which people discuss particular weather phenomena varies in accordance with environmental factors (Kemp et al., 2018). Less obviously, urban and industrialized environments yield an increased likelihood that certain colors are foregrounded and discussed, yielding an apparent influence on the development and usage of some color terms. Evidence suggests that languages spoken by industrialized groups tend to develop more precise color terms for brightly colored hues associated with modern techniques of dying and coloring (Gibson et al., 2017). Given that agriculture and industrialization are not stochastically associated with environment types, such factors hint at indirect environmental influences on speech. The kinds of spatial language speakers employ are impacted more directly by the environments in which they are embedded, as evidenced for instance by experimental research in virtual environments (Nölle et al., 2018). Combinations of certain lifestyle types in particular ecologies may also impact the likelihood that speakers come to use robust sets of abstract terms for odors (Majid et al., 2018). These are just some of the ways in which environmental factors appear to influence lexical phenomena.

With respect to phonetic and phonological phenomena, research suggests that the diet types characteristic of particular cultures can impact the likelihood that the members of those cultures use particular sound types. Languages spoken by people with softer diets are more likely to rely on labiodental consonants, presumably because the softer diet yields characteristic overbite and overjet dental configurations in adults (Blasi et al., 2019). These configurations, in turn, yield a greater ease of articulation of labiodental consonants. Given that softer diets are largely a byproduct of agriculture of particular kinds, this fact hints at a long-term probabilistic yet indirect effect of physical environments on speech [Of course, the degree to which cultures rely on agriculture is due to a complex interaction of factors including environment and cultural transmission patterns (Vilela et al., 2020)]. The fact that labiodental consonants are associated with particular bite types has now been supported by a range of findings, including biomechanical modeling, diachronic trends, phonological typology, the frequency of sounds in wordlists worldwide, and the observation of the phonetic tendencies of individuals with divergent bite types (Blasi et al., 2019; Everett and Chen, 2021).

Related research has also suggested that the ambient characteristics of given cultures impact in more direct ways, though subtle and gradual ones, the extent to which their languages rely on certain kinds of sounds. More specifically, it has been hypothesized that extremely arid climates, most notably those in very cold regions with typically low specific humidity, place pressures on the ease of articulation of certain laryngeal gestures required for complex tonality and vowel production (Everett et al., 2015; Everett, 2017). While more direct, these putative environmental effects would nevertheless surface crosslinguistically via well-established diachronic and sociolinguistic phenomena (Everett, 2021). The central claim in such work is that some phonetic phenomena might be triggered at slightly different rates due to very minor variations in the ease and precision of vocal cord vibration, owing to the effects of aridity on the vocal cords’ viscosity (Leydon et al., 2009). Ease of articulation is already well known to impact the rate at which certain sound types occur in speech and in phoneme inventories worldwide, so the central mechanism at the heart of this hypothesis is itself uncontroversial. Nevertheless, it is unclear whether environmental factors like extreme aridity impact ease of production of the relevant articulatory gestures, at least to the extent that they subtly influence diachronic sound changes, and some objections have been raised to this hypothesis (e.g., Collins, 2016). In short, while correlational data are broadly consistent with the possibility of a direct ecological effect, the likelihood of this possibility is contested. Setting aside these particular debates about direct long-term ecological effects on sound use, there is growing consensus that languages are affected indirectly and directly by environmental factors in ways that have only recently been considered (Bentz et al., 2018).

While environmental factors may impact the way that languages evolve over the long-term, speech can conversely impact the immediate environment in invisible and inaudible ways. As people speak, they do not simply emit energy via the propagation of sound waves. They also emit air molecules and particles, including aerosolized particles. Aerosol particles are suspended in the air and often defined as ranging in size from 10 nm to 5 μm in diameter. Particles larger than this (i.e., droplets) are also generated during speech, as described in the literature (e.g., Stadnytskyi et al., 2020). Although 5 μm is often used as a cut-off to distinguish aerosols from droplets, a size of ~100 μm should be considered as an alternative cut-off as this figure denotes the largest particle size that can remain suspended in still air for more than 5 s from a height of 1.5 m (Wang et al., 2021; Darquenne et al., 2022). Our focus here is on the airflow and aerosol particles generated during speech. In the following section, we describe a new method developed for simultaneously capturing acoustic, airflow, and aerosol particle data during speech. In the remainder of this section, we offer some relevant background from the literature on the production of airflow and aerosols.

Humans produce air molecules, including carbon dioxide, oxygen, and nitrogen, during expiratory activities like speaking and singing. These molecules are only a fraction of nanometers in size, but are exhaled in tremendous volume with airflow. There are numerous findings in phonetics and biomedicine demonstrating how certain kinds of articulations yield varying amounts of airflow. We focus here on the airflow findings related to consonants in English, as this is relevant to our subsequent discussion of aerosol particles. Vowels typically have limited peak airflow, and there is little variation in peak airflow between vowels (Baken and Orlikoff, 2000, chapter 9). More specifically, we focus on key results in the literature related to the peak airflow of word-initial and word-final consonants, as measured in mL/s. It is important to note that airflow varies substantially according to body size and lung capacity, at least in the case of egressive pulmonic consonants. Stathopoulous (1980) examined the airflow associated with consonant production in English-speaking adults, teenagers, and children. Adults were found to produce significantly greater airflow across the same consonant types, with teenagers producing greater airflow than younger children. The findings were based on word-initial and word-final consonants, and clear patterns also emerged across consonant types. Nasal consonants were not included in the analysis, which focused on oral airflow. The consonants associated with the lowest peak airflow were word-final voiced stops and fricatives. Voiceless plosives and fricatives, particularly in word-initial contexts, were associated with greater peak airflow. The reduced airflow associated with voiced consonants is due in part to the blockage of the airstream at the glottis during vocal cord vibration, which limits peak egressive airflow. This same factor limits the peak airflow of vowels.

**Figure 1:**
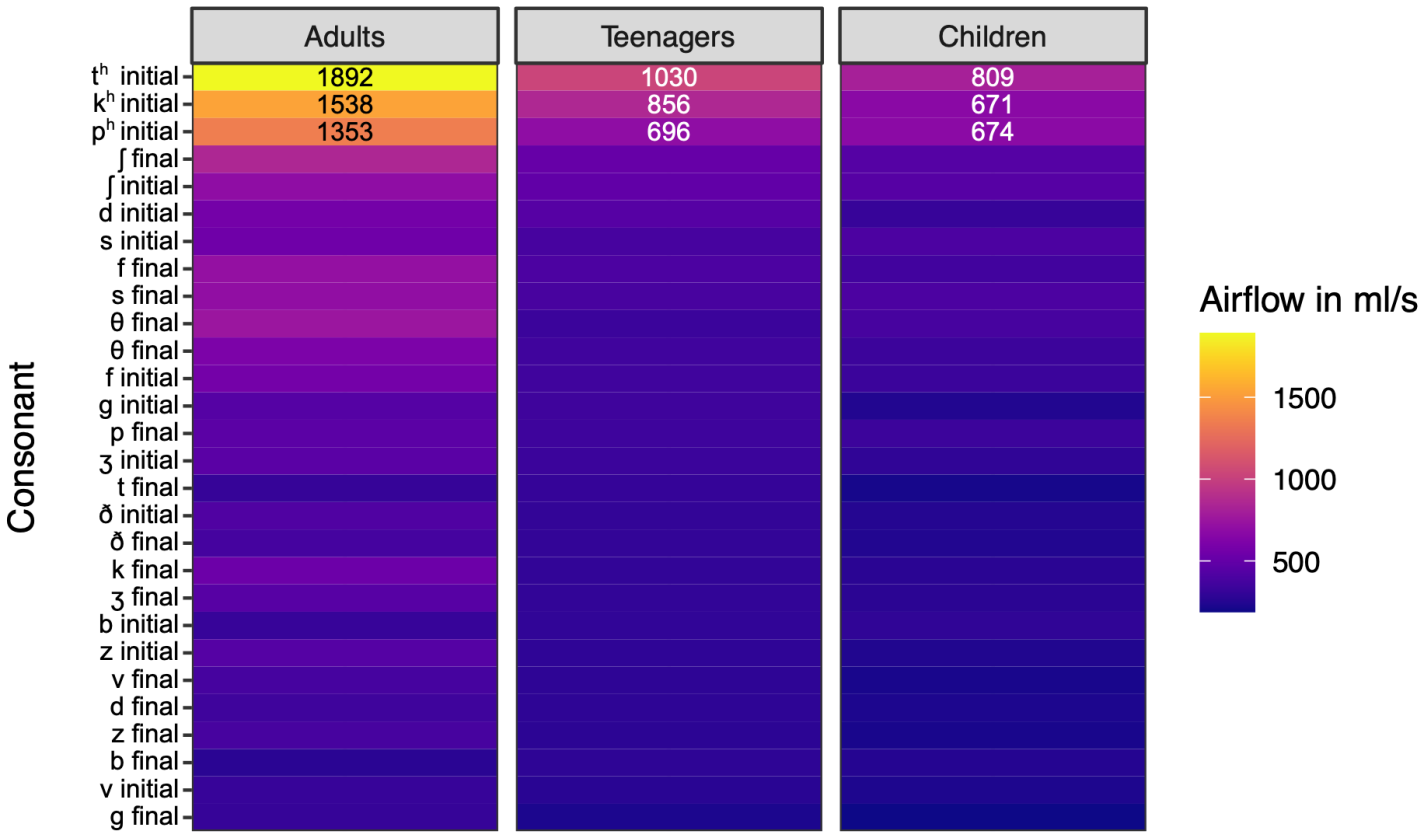
Heatmap of peak airflow associated with plosives and fricatives in English. Based on data in Stathopoulous (1980). The top three airflow values for each speaker category are provided on each of the appropriate bars.

In Figure 1, we offer a visualization of peak airflow across key English consonants, based on relevant data in Stathopoulous (1980). In the figure, the greater peak airflow associated with word-initial voiceless consonants, in particular word-initial aspirated plosives, is readily apparent. These data are based on averages for 10 adults (five male), 10 teenagers (five male), and 10 children (five male). Note that the aspirated consonants of adults yield peak airflow up to three times greater than that evident in other consonants tested, with the mean peak airflow exceeding 1,700 mL/s. Given the average adult male lung vital capacity is roughly 6 L; this suggests that a significant portion of pulmonic air can be used during the production of aspirated consonants. The anomalous nature of aspirated consonants is also evident in our airflow data, some of which are presented below. It is worth noting that, while common in English, aspirated consonants are not particularly frequent cross-linguistically. This is supported by an inspection of PHOIBLE, the most extensive database on phoneme inventories worldwide (Moran and McCloy, 2019). Judging from the 3,183 phoneme inventories represented in PHOIBLE, [t^h^] is found in fewer than one fifth of the world’s languages. It is found in 17% of inventories, while [p^h^] and [k^h^] are slightly more prevalent, each occurring in roughly 20% of inventories. [p^h^] is found in 592, while [k^h^] is slightly more common, being documented in 605. While not particularly frequent cross linguistically, these sounds are hardly typological rarities either. Intriguingly, it has been speculated that aspirated consonants may be associated with greater likelihood of airborne pathogen transmission during speech (Inouye, 2003). While this remains a speculation, the approach we present in the next section allows for the detection of both airflow and aerosol particles, which can potentially host pathogens, offering a less speculative route to the future exploration of this and other related issues.

While research on the airflow associated with the production of speech dates back decades, only in the last few years have studies begun to emerge that address the aerosol particles produced during speech. New devices allow for the detection of aerosol particles, though such devices are generally applied to nonlinguistic phenomena. They can, however, be adapted to explore the production of aerosols during speech. Speech-based aerosols have received increased attention in the last several years due to the advent of such devices and associated instrumental adaptation, and also due to the fact that it became increasingly clear that such speech-based particles were relevant to the transmission of the SARS-CoV-2 virus among asymptomatic individuals (Abkarian et al., 2020; Fennelly, 2020; Meselson, 2020). Case studies demonstrated early in 2020 that speakers and singers could transmit this virus, yielding a push to better understand the mechanisms through which humans produce viral-laden particles during speech (Hamner et al., 2020; Bahl et al., 2021). That push remains underway, and a variety of methods are being deployed to better illuminate how exactly aerosols are generated during the articulation of sounds. These methods include the utilization of laser sheets and aerodynamic particle sizers to isolate the size distribution of miniscule particles produced during specific articulatory gestures (Stadnytskyi et al., 2020). Work relying on an aerodynamic particle sizer (APS) has suggested, for instance, that the high front vowel /i/ yields an inordinate number of aerosol particles when contrasted to other phonemes in English (Asadi et al., 2020). More critically, APS-based research has suggested that the volume of aerosol particles produced during speech is a function, at least in part, of the amplitude at which the vocal cords vibrate (Asadi et al., 2019). Judging from such work, vocal cord vibration appears to be the chief mechanism through which aerosols are produced during speech. There are two key caveats to this conclusion, however. First, work to date has not simultaneously examined airflow, aerosol, and acoustic data. Instead, the conclusion has been based on research demonstrating an association between increased amplitude of vocal cord vibration and aerosol production. Given that increased amplitude of vocal cord vibration is achieved partially through greater airflow through the glottis, such an approach makes it difficult to disentangle the relative contributions of amplitude and airflow. The approach we outline below allows for such disentanglement since it includes simultaneous measures of airflow, aerosol, and acoustic data. A second caveat associated with the relevant conclusions in the literature, vis-à-vis the association of sounds like /i/ and increased aerosols, is that they rely on a method with limited temporal resolution. The APS used in such studies samples air once per second. Since words, syllables and in particular phonemes typically last less than 1 s, this means that the method requires the repetition of stimuli over a particular duration, during which time the total number of aerosols is measured (Greenberg et al., 2003; Asadi et al., 2020). This number of aerosols is then correlated with the number of particular sound types, for instance /i/, in a given set of phonetic stimuli. Thus, testing aerosols once per second does not allow for the direct observation of the production of aerosols during specific articulatory gestures. In part for this reason, we developed an approach with greater temporal resolution, one that allows us to sample air 10 times per second, to more confidently make assessments regarding the role of individual articulatory gestures in aerosol production. Such heightened physical resolution is critical to better isolating the extent to which vocal cord vibration or alternate mechanisms actually produce aerosols. We return to this point below. Our approach also allows for a greater physical resolution, with the potential to observe aerosols with diameters as small as 70 nm, or about the size of some airborne viruses. Previous approaches generally allow only for the isolation of those particles greater than 500 nm in diameter (Morawska et al., 2009; Asadi et al., 2020). Some airborne virions, which are infective forms of viruses, can be hosted by particles as small as 90 nm in diameter, so capturing particles in this size range is potentially relevant to speech-based viral transmission (Lee, 2020).

More broadly, the approach we describe could eventually help to impact public health guidance related to speech during future airborne pandemics. Some widely disseminated guidance in 2021 suggested that people should reduce vocal cord vibration via whispering, in order to reduce the risk of transmitting the SARS-CoV-2 virus (Thompson, 2020). As we will see below, further work is needed to support such guidance and some of our preliminary findings are inconsistent with this suggestion. Relatedly, there has been some speculation in prominent venues like The Lancet that consonant aspiration could help to transmit airborne viruses (Inouye, 2003). We avoid such speculations here, though we return to aspiration below as our preliminary results suggest that it produces a greater number of aerosols alongside the increase in peak airflow. Such results, while quite preliminary and requiring caution to interpret, demonstrate that exploration of this understudied topic could help to elucidate our understanding of airborne disease transmission during speech. While air molecules do not transport pathogens, aerosol particles that can do so are suspended within that airflow (Wang et al., 2021). Characterizing these aerosolized particles is key to quantifying and modeling respiratory pathogen transmission risk, especially since small particles (<3 μm) penetrate deeper into the lung and infection in the lower respiratory tract requires fewer numbers of pathogens to produce lethal infection in animal models (Thomas, 2013). Additionally, depending on the primary mode of transmission of an infectious respiratory pathogen, understanding the size of particles produced during speech can have significant implications on use and effectiveness of non-pharmaceutical interventions for transmission mitigation in an outbreak setting (Leung, 2021). The first step in this elucidation is, in our view, to illuminate in greater detail the actual articulatory mechanisms through which airflow and aerosols are produced. Regardless of its potential eventual influence on our understanding of airborne pathogen transmission, however, this illumination will allow us to better understand the invisible effects of speech on the proximate physical environment. In the following section, we discuss this new approach, illustrating how it allows for the isolation of the aerosols produced by both aspiration and vocal cord vibration.

## 2. Examining the phonetic production of airflow and aerosols via a new approach

In this section, we first offer some new data on airflow, which is relevant to contextualizing our approach. We then describe the method being used to analyze airflow, aerosol, and acoustic data simultaneously. Finally, we offer some very preliminary data with this approach, based on the speech of two of the authors. These preliminary data demonstrate how the method allows for the isolation of the role of individual articulatory gestures in the production of aerosols. Further, the preliminary data suggest that aspiration produces an inordinate number of aerosol particles below the threshold of detection of previous methods.

**Figure 2:**
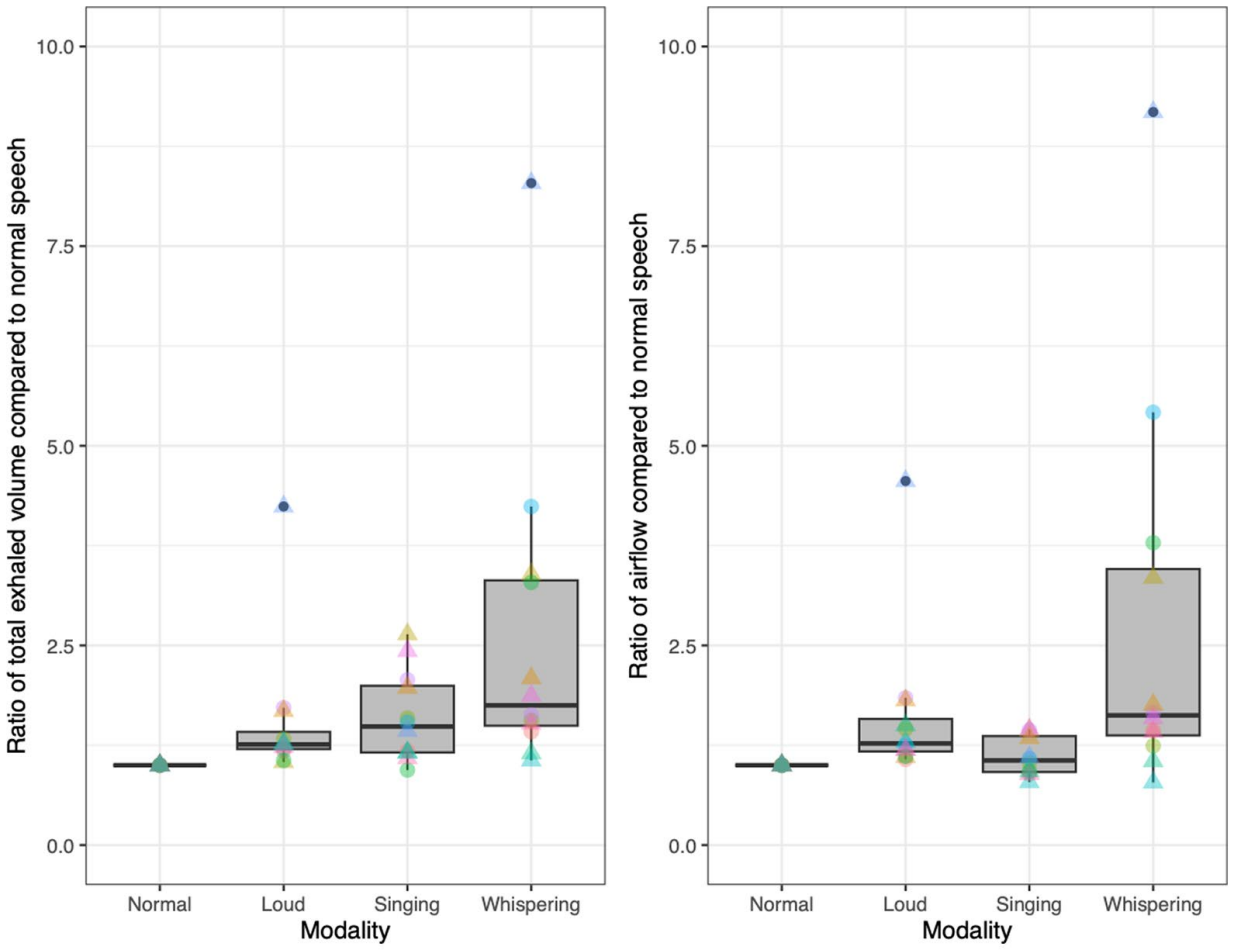
Normalized exhaled volumes **(left)** and airflow **(right)** across modalities for 12 speakers (six male), based on each speaker’s exhaled volume/airflow as a ratio of their mean exhaled volume/airflow during normal speech. Triangles represent male speakers. Each color corresponds to an individual.

We analyzed the airflow of 12 fluent English speakers (six male), to better contextualize our examinations of aerosol production. To do so, speakers wore a mask connected to a pneumotachograph (Fleisch no. 1, OEM Medical, Richmond, VA, United States) to record flow as they sang “happy birthday,” but also as they whispered “happy birthday” and as they spoke the words to the song, at a normal amplitude and at a loud amplitude. Mean flow rate and exhaled volume were averaged over four repetitions of the song for each modality. During normal speech, speakers produced an average of 150 mL/s of airflow and exhaled an average of 1.2 L of air throughout “happy birthday,” though there was variation across speakers as we might expect. Mean airflow and exhaled volume across speakers was 157 ± 42 mL/s and 1,204 ± 339 mL [average ± standard deviation (SD), *N* = 12]. In Figure 2, we present the normalized mean airflow and exhaled volume across modalities. In the figure, each speaker’s normal speech airflow and exhaled volume are set to one and the other modalities are presented as a ratio of the airflow and exhaled volume to that of normal speech, respectively. Four of the speakers exhibited a pronounced increase in airflow and exhaled volume of air during whispering, with one speaker producing nine times the flow rate and eight times the exhaled volume as he did while speaking at a normal volume. Another subject produced five times the flow rate and four times the exhaled volume during whispering when compared with normal speech. Whispering involves a constricted glottis without vibrating vocal folds, so airflow is not regularly blocked as it is with sounds like vowels (Sundberg et al., 2010). This point is relevant to the production of aerosol particles. There are several potential mechanisms for the production of such particles in the respiratory tract. Two of these are particularly relevant to this discussion. One involves a fluid-film burst in the bronchioles, which creates aerosols that can then be emitted. The larger the exhaled volume is the greater the number of exhaled aerosols and thus the greater the concentration in the surrounding environment. Aerosols originating deep in the respiratory tract via this mechanism may have a greater likelihood of transmitting viral pathogens (Lindsley et al., 2016). A second relevant mechanism for aerosol generation is the vibration of the vocal cords, the viscous covering of which can burst into particles including tiny aerosol particles. The higher the exhaled flow rate is, the higher the shear stress and the greater the aerosol generation. This mechanism is presumably responsible for the increased aerosols associated with vowels, particularly loud vowels, in the literature (Asadi et al., 2019). However, as noted above most studies in the literature did not detect particles smaller than 500 nm in diameter.

**Figure 3:**
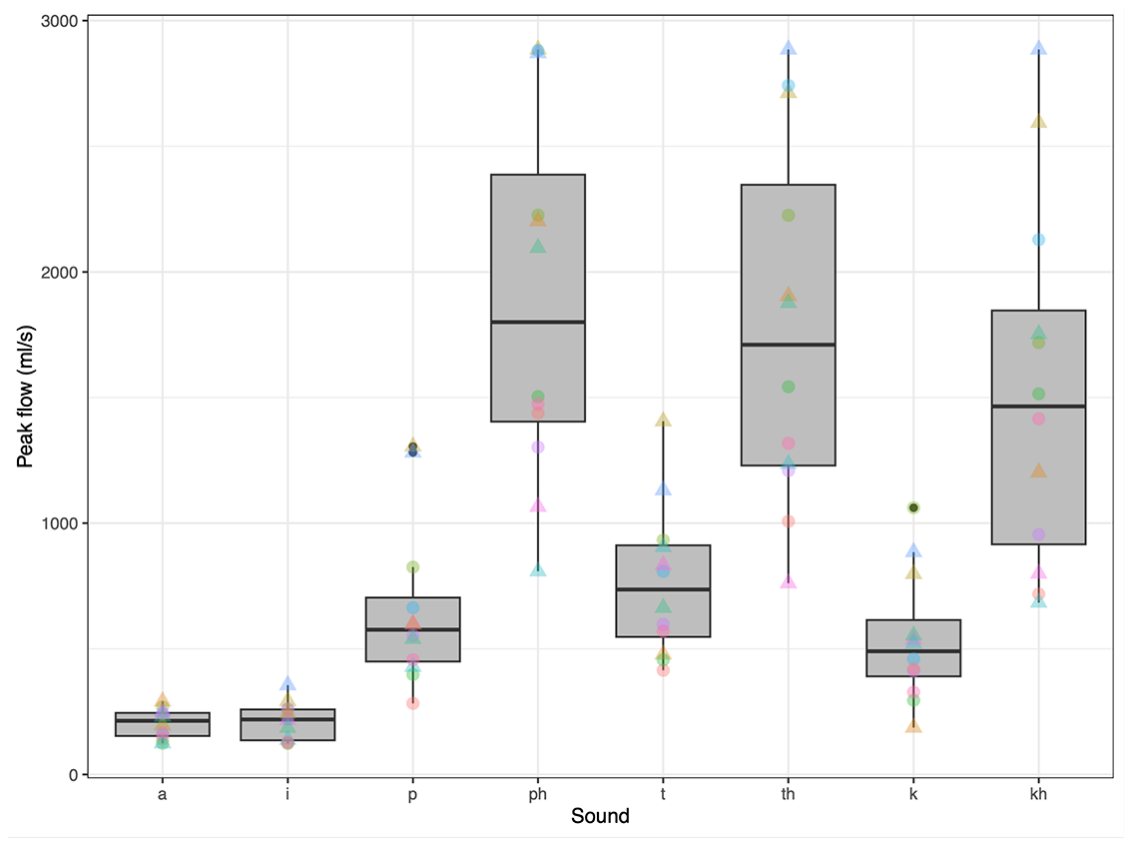
Peak airflow associated with two English vowels and six voiceless plosives, for 12 adults (six male). Triangles represent male speakers. Each color corresponds to an individual.

For this background airflow analysis, we also recorded the speakers as they produced individual words and two vowels, [a] and [i], at a normal amplitude. Three pairs of words were recorded: (1) “spar” and “par,” (2) “star,” and “tar,” and (3) “scar” and “car.” For each of these pairs, the first word includes an aspirated plosive while the second includes a non-aspirated version of the same voiceless plosive, i.e., made at the same place of articulation. As apparent in Figure 3, the peak airflow associated with aspirated voiceless plosives was noticeably greater than that associated with non-aspirated plosives, consistent with Figure 1. This increase was observed across all 12 speakers and at each place of articulation. The mean peak airflow across all speakers was greatest for the aspirated voiceless bilabial stop, with a mean that exceeded 1,800 mL/s. The two vowels tested produced negligible peak airflow (means <100 mL/s).

This context on the airflow associated with whispering and aspiration is useful to our ongoing exploration of the aerosols produced during speech. Since the airflow associated with whispering and aspiration is pulmonic and since neither whispering nor aspiration entail voicing, it is expected that any aerosols detected during such speech activities are due to the fluid-film burst mechanism, originating from deep within the respiratory tract. Further, the quantification of the airflow associated with voiced sounds like [a] and [i] helps to illuminate the extent to which aerosols observed during the production of such sounds are due directly to vocal cord vibration, or potentially due to the increased airflow associated with greater amplitude of vocal cord vibration. As observed in Figure 2, there is typically an increase in the mean airflow for loud speech, when compared to speech at a normal amplitude. As noted above, this complicates the interpretation of the results in the literature suggesting that the aerosol increase associated with loud vowels is due in a straightforward manner to the increase in the amplitude of vocal cord vibration as opposed to airflow carrying aerosols from deeper within the respiratory tract.

**Figure 4:**
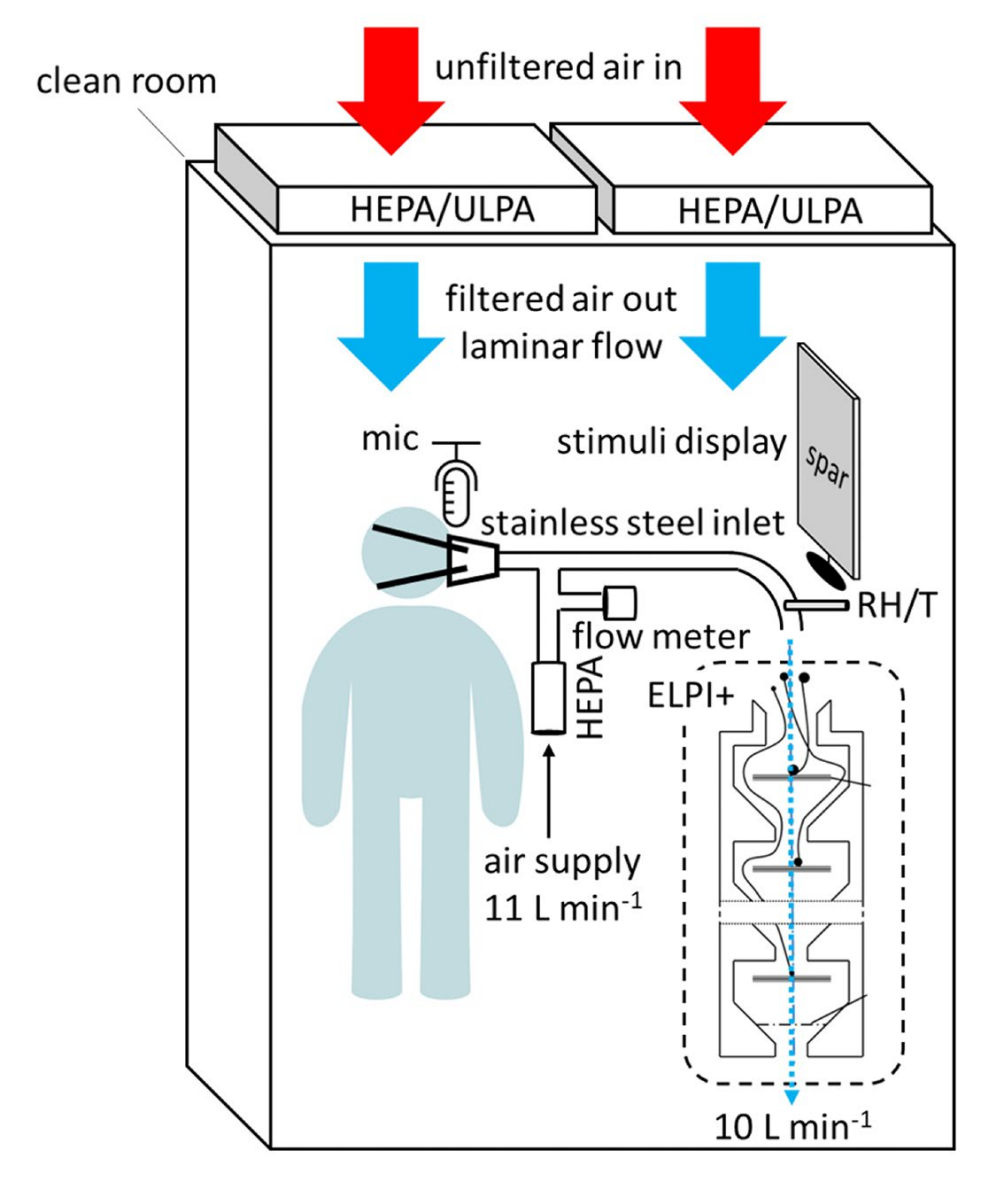
Schematic of new method for simultaneously capturing data on airflow, sound types, aerosols, temperature, and humidity.

**Figure 5:**
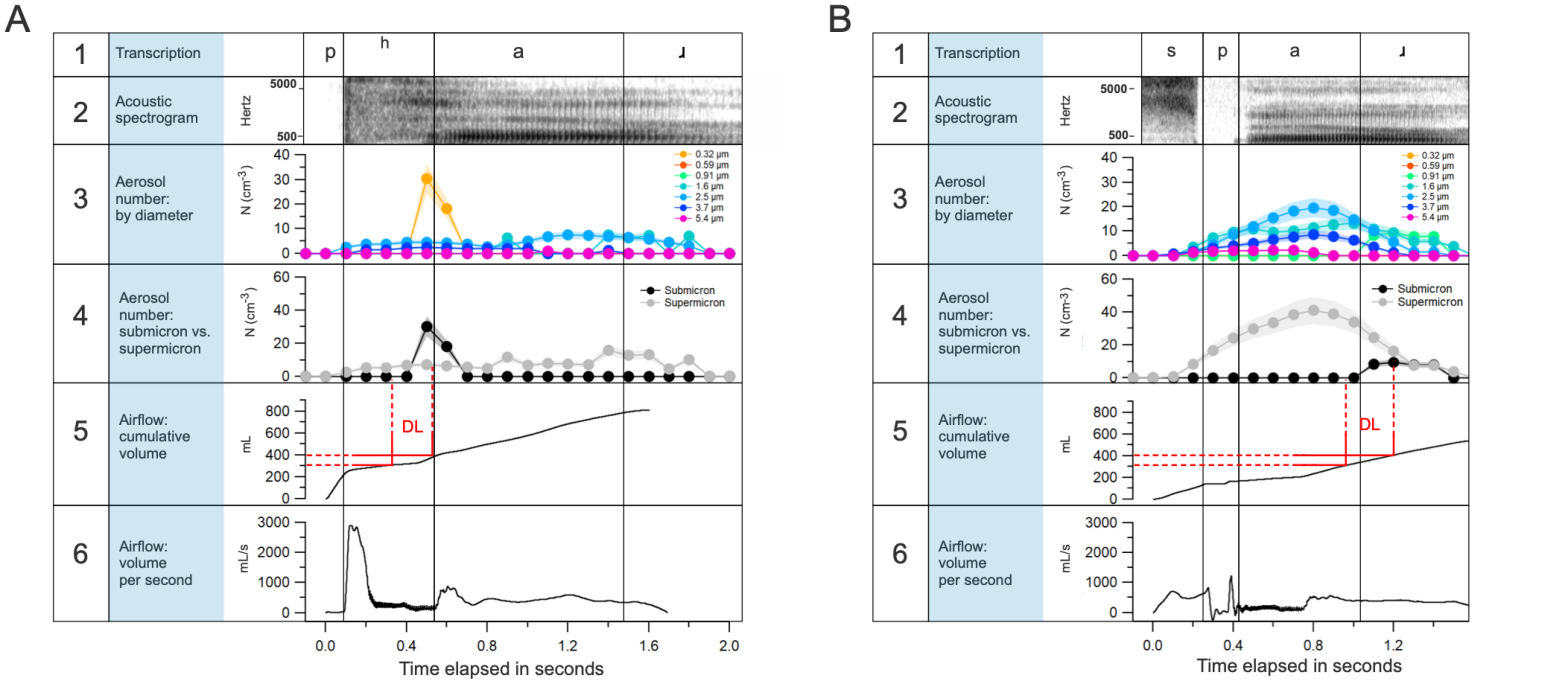
Temporal resolution allowed by the method. Aerosols as naturally produced during a speaker’s articulation of “par” **(A)** and “spar” **(B)** with spectrogram, airflow, and aerosol data offered simultaneously. In A5 and B5, “DL” refers to the approximate range of cumulative volume of air over which air from the “deep lung” gets emitted and corresponds to the increase in submicron respiratory aerosol concentration in A4 and B4, respectively.

This background on airflow associated with both aspiration and vocal cord vibration serves as critical contextualization of our discussion of the aerosol production owing to these key articulatory gestures. Here we focus on these gestures to illustrate our new method for simultaneously capturing aerosol, airflow, and acoustic data. Ongoing research utilizing the method is exploring aerosol production with a large number of speakers in the lab of the last author. Previous work has simultaneously examined airflow and acoustic data (e.g., Yu et al., 2022), but no studies to date have illustrated a method capturing these data alongside aerosol data. The method we have developed is described schematically in Figure 4. Experiments proceed as follows: Participants sit alone in a mini clean room surrounded by a downward laminar flow of HEPA-filtered air, which creates an environment that is nearly free of background aerosols. They then read prepared stimuli off of a screen, into a rubber mask that is attached to their mouths. The rubber mask leads directly into a custom-built stainless steel particle sampling manifold, which curves gently into an electrical low-pressure particle impactor (ELPI+, Dekati Ltd.) that measures aerosols from 70 nm to 10 μm in size (Järvinen et al., 2014). Details of this particular ELPI+ are provided in Tumminello et al. (2021). Pure air is fed into the manifold at a rate of 11 L per minute. A flow meter detects fluctuations in this airflow resulting from the incoming airflow generated by the speakers. Above the facemask, there is a microphone which records audio stimuli directly to a laptop computer at 44.1 kHZ, via PRAAT (Boersma and Weenink, 2023). As the vacuum pump necessary for ELPI+ operation is not quiet, the resultant waveforms and spectrograms do include some background noise. Given that our present focus requires only coarse acoustic data to interpret key articulatory gestures, this does not present an issue, particularly given that the airflow data yield clear signatures for vocal cord vibration and aspiration (see Figure 5). For future analyses with more acoustic detail required, we aim to use sound proofing materials in the setup. It is also worth noting that the relative humidity and temperature of the air leading into the ELPI+ is measured, allowing us to test the effect of humidity on the number distribution of aerosol particle sizes. Humidity is well known to affect the ways that speech-generated particles interact with the surrounding air (De Oliveira et al., 2021).

Upon entering the ELPI+ inlet, the speech aerosol particles are initially charged with a positive corona charger before traveling down through the impactor. The unipolarly charged particles are then collected at each impactor stage on high surface area sintered plates, which are coated with a thin layer of high viscosity vacuum grease to maximize collection efficiency. Particles are size segregated by their aerodynamic diameter over 14 stages, ranging from 10 μm at the inlet to 5 nm at the bottom stage of the impactor. Particle collection is measured by sensitive electrometers (fAmp sensitivity) on each stage at a sampling rate of 10 Hz. The resulting currents are converted to number concentrations based on particle size.

**Figure 6:**
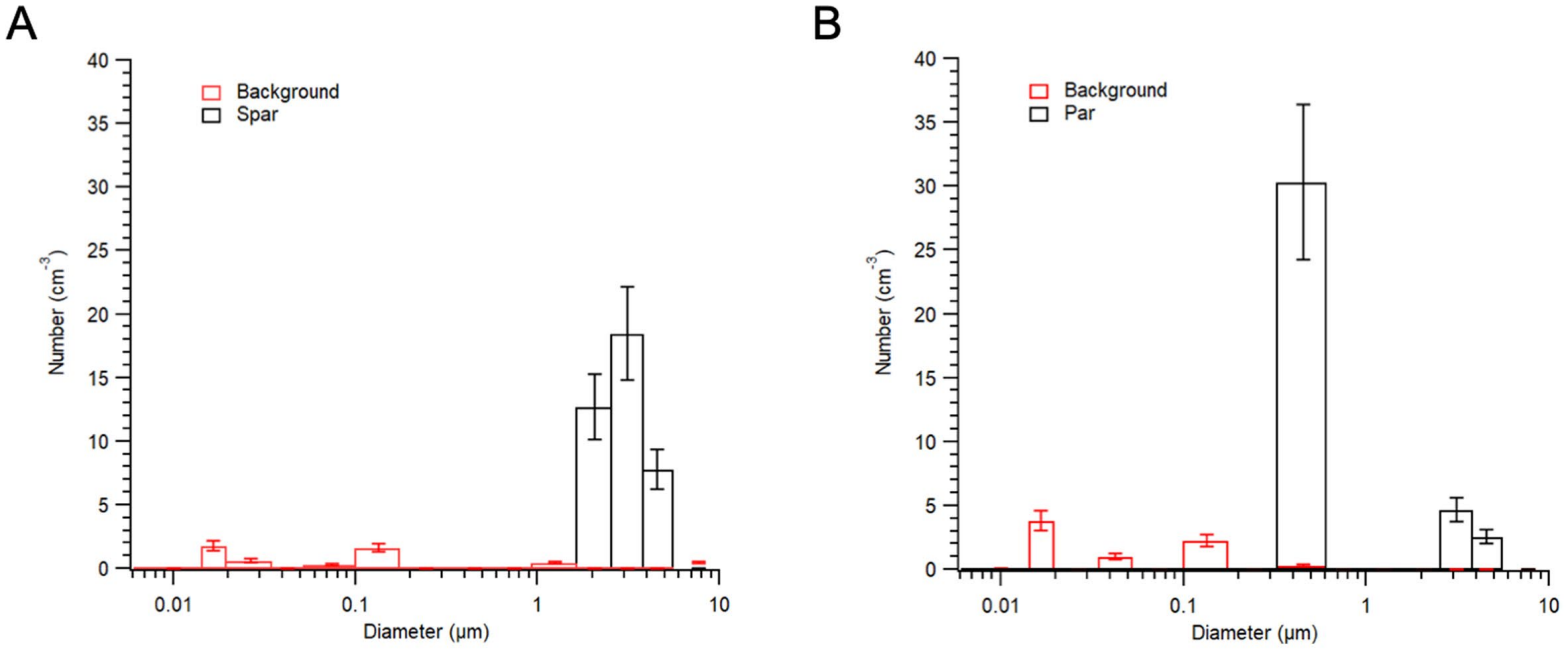
Physical resolution of the method, as evidenced by aerosols detected during one speaker’s articulation of “spar” **(A)** and “par” **(B)**. Note that during the speaker’s productions of the word “par” there was a particularly pronounced increase in submicron particles, likely due to the aspiration of the first sound of the word. All submicron aerosol data in red (background) are below instrumental detection limit and cannot be attributed to aerosol.

Across both speakers whose aerosols have been measured without background particles (both males), we have found that aspiration is associated with an increase in the production of submicron particles. Given that we have only tested two speakers with this method, we stress that these results are meant only to illustrate the enhanced physical and temporal resolution of our method. In Figure 6, the physical resolution of the method is demonstrated. Based on averages of five iterations each of the words “spar” and “par,” we see that the word “par,” beginning with a voiceless aspirated bilabial plosive, is associated with an increase in aerosol particles with diameters of around 300–500 nm. Note that such particles were not detectable in most previous studies relying on an APS, which is limited to particles greater than 500 nm. Further, we see in Figure 6 that speech produces dozens of aerosol particles in the case of both words, while the background particles are nearly nonexistent or below the instrumental detection limit in the clean room environment. Nevertheless, there are some background particles and these fluctuate slightly under the laminar hood. This is evidenced by the slight differences in the red lines for panels A and B in Figure 6. Note also that there is some variation in the number of larger particles (diameter > 1 μm) produced during the words “spar” and “par” in these instances. These variations could be due to slightly louder productions of the vowel in the word “par,” or to random fluctuations for these particular instances of these words. We stress that these results are preliminary and that we aim to run these tests with many individuals and sound stimuli prior to drawing conclusions about the associations between particular sound types and their associated aerosols. This will be necessary to reduce the effect of noise in the data, but also to reduce the undue influence of idiosyncratic findings associated with individual speakers.

The method offers a more critical advantage for exploring the invisible effects of speech on the environment: It allows for fine-grained temporal resolution given the 10 Hz sampling capacity of the ELPI+. In Figure 5, this temporal resolution is illustrated via an analysis of the first author’s deliberate articulation of two words, “par” and “spar.” As evident in panel A of the figure, there is a peak in submicron aerosols immediately after the burst of airflow owing to the aspirated bilabial plosive in “par.” This aerosol burst coincides with the point at which the cumulative exhaled volume exceeds 300–400 mL, which is consistent with work suggesting that tiny aerosols generated deep in the lungs are emitted from volumetric depths beyond the anatomical dead space (i.e., volume of air in airways down to the respiratory bronchioles) during expiratory activities (Gebhart et al., 1988). A similar pattern is observed in panel B, but note that the 400 mL threshold is achieved much later in the word due to the lack of aspiration in the word “spar.” In panels A and B, we observe that larger aerosol particles, greater than 1 μm in diameter, are generated shortly after the vocal cords begin to vibrate, as evident in the alignment with the spectrogram. This is consistent with the literature that has focused on vocal cord vibration as a source of larger aerosol particles. Our preliminary results suggest, then, that the two aforementioned potential loci of the origination of speech-generated aerosols, the vocal cords and the bronchioles, are detectable and isolated via our method. That is, it appears we are able to detect when aerosols are generated at the glottis during vocal cord vibration, and when they are generated deep within the respiratory tract and emitted alongside airflow such as that characteristic of aspiration. Of course, we need much more data before offering any conclusions on the role that individual articulatory gestures play in aerosol production. To that end, future work will test dozens of English speakers to more carefully isolate the roles that consonant aspiration and vocal cord vibration play in generating aerosol particles during speech.

Finally, while we think this method represents a step forward in terms of how we might investigate the precise mechanisms through which speech generates aerosols, we also recognize that the approach has limitations and should be complemented by other approaches. One limitation is that speakers must wear a tight-fitting mask during the tests and must face the same direction during the whole test. Similarly, the equipment used is not quiet, so speakers may compensate by increasing their loudness to more clearly hear themselves speak. In short, while the method offers advances it does not allow us to test the aerosols produced in natural conversation-like settings. No method available to date allows this. We should also mention that this work is limited in that we are only examining English speakers at present. In the future we hope to test speakers of other languages.

## 3. Conclusion

We began this paper by discussing some of the proposed invisible effects of the environment on how people speak. We then focused our discussion on the converse issue that has received even less attention in language research: the invisible and inaudible effects of speech on the immediate environment. This topic offers two key gains, when contrasted to the exploration of the ways in which languages are affected by their environments. First, the topic can be addressed more directly via experimentation, though that experimentation presents a number of challenges and requires costly equipment. Second, exploration of this topic has the potential to do more than shed light on the nature of language and its relationship to the physical environment. Such exploration may ultimately yield health guidance related to speech that is firmly founded on a clearer understanding of how sounds generate potentially viral laden aerosol particles. In short, the issue has potential relevance not just to our understanding of speech, but perhaps to contemporary medicine as well. The precise articulatory mechanisms that help transmit pathogens during conversations are still not fully understood, but hopefully that will change in the coming years. Here we have described a new method that could assist in the elucidation of those mechanisms.

## Data availability statement

The original contributions presented in the study are included in the article/supplementary material, further inquiries can be directed to the corresponding author.

## Ethics statement

The studies involving human participants were reviewed and approved by UCSD IRB. The patients/participants provided their written informed consent to participate in this study.

## Author contributions

CE, MS, CD, and JS conceptualized, funded, and supervised the study. CE wrote the original manuscript draft and analyzed the acoustic data. CE, MS, RN, PT, CD, and JS reviewed and edited the manuscript. PT, RN, and JS performed the aerosol measurements and analyzed the aerosol data. CD performed the volume flow measurements and analyzed the volume flow data. CD, CE, MS, JS, PT, and RN collected the data. All authors contributed to the article and approved the submitted version.

## Funding

CD acknowledges funding by National Institute of Health grant U01 ES028669. PT and JS acknowledge funding support by the National Science Foundation Center for Aerosol Impacts on Chemistry of the Environment under Grant CHE-1801971. RN acknowledges support by a National Science Foundation Graduate Research Fellowship.

## Conflict of interest

The authors declare that the research was conducted in the absence of any commercial or financial relationships that could be construed as a potential conflict of interest.

## Publisher’s note

All claims expressed in this article are solely those of the authors and do not necessarily represent those of their affiliated organizations, or those of the publisher, the editors and the reviewers. Any product that may be evaluated in this article, or claim that may be made by its manufacturer, is not guaranteed or endorsed by the publisher.
